# 

**Published:** 1878-02

**Authors:** 


					To Readers and Correspondents.
Communications intended for publication, and exchange journals and papers
should be addressed to the Editor of the American Journal of Denta
Science, 259 N. Eutaw St., Baltimore, Md. All letters relating to the busines
of the journal, or containing cash remittances, to be directed to Messrs. Snowde
& Cowman. Articles for publication should be received by the editot at leas
three weeks previous to the first day of the month on which the number in whic
it is designed for them to appear, is issued.
I^WThe American Journal of Dental Science will contain tiftj pages of
reading matter in each number, making a volume of about
Six Hundred pages
EXCLUSIVE OF ADVERTISEMENTS
THE
AMERICAN JOURNAL
OF
DENTAL SCIENCE.
The Journal is issued on the first of each month and contains not less
than fifty pages of reading matter in each number, exclusive of adver-
tisements, making a volume of six hundred pages per annum.
In each number will be found Original Communications, Corres-
pondence, Selected Articles, Monthly Summary, Bibliographical No-
tices and Editorial Matter, and every effort will be exerted to render
the Journal worthy of the patronage of the Dental profession.
A generous co-operation is respectfully solicited from the members
of the profession ; and as the Journal has now a printing office of its
own, which materially lessens the cost of its publication, it will be
issued at the reduced subscription price of
92.SO Per .Annum in Advance.
Communications are respectfully solicited on all subjects pertain-
ing to the practice of dentistry, as well as reports of cases occurring
in dental practice.
RATES OF ADVERTISING.
1 Page.
* "
\ "
1 MO.
$15 00
10 00
7 00
5 00
2 MO.
$22 50
15 00
11 00
8 00
3 MO.
$30 00
20 00
15 00
11 00
6 MO.
$50 00
30 00
20 00
15 00
12 MO,
$100 00
50 00
30 00
20 00
All original communications designed for publication, works for
review, new instruments for notice, exchanges, &c., should be directed
to the editor, 259 N. Eutaw St., Baltimore, Md.
All advertisements and subscriptions should be addressed to
SNOWDEN & COWMAN,
Publishers of the Am. Journal of Dental Science.
86 W. Fayette St. Bet. Charles & Liberty Sts.,
BALTIMORE, MD

				

